# Educational and Personal Opportunity Costs of Medical Student Preparation for the United States Medical Licensing Examination Step 1 Exam: A Single-Center Study

**DOI:** 10.7759/cureus.10938

**Published:** 2020-10-14

**Authors:** Nicolas W Cortes-Penfield, Rohan Khazanchi, Geoffery Talmon

**Affiliations:** 1 Department of Internal Medicine, University of Nebraska Medical Center, Omaha, USA; 2 School of Public Health, University of Minnesota, Minneapolis, USA; 3 Department of Pathology and Microbiology, University of Nebraska Medical Center, Omaha, USA

**Keywords:** usmle, step 1, score, medical student education, nmbe, usmle step 1, step 1 score, medical education, residency application process, residency preparation

## Abstract

Purpose

To assess the degree to which medical students choose to disengage from their regular preclinical curriculum and extracurricular activities in order to focus on United States Medical Licensing Examination (USMLE) Step 1 exam preparation, as well as learner-perceived effects of Step 1 preparation on their physical, social, and mental health.

Method

Online survey of medical students who have taken the USMLE Step 1 exam at a single large Midwestern academic medical center.

Results

The response rate was 54%. Students often reported absenteeism from a variety of preclinical curricular activities, including lectures (44%) and didactics focusing on medical ethics (37%), clinical skills (28%), and encounters with actual and standardized patients (9%) in order to study for USMLE Step 1. Many students also forewent extracurricular opportunities including research (53%), elective patient care opportunities (45%), community service (39%), and healthcare advocacy experiences (38%) in order to study for USMLE Step 1. Majorities of students identified Step 1 preparation as a cause of burnout (79%) or significant anxiety or depression (61%), for which nearly a third sought mental healthcare; students also reported Step 1 preparation as a cause of engaging in dangerous behaviors such as illicit prescription stimulant use as well as driving or providing patient care while impaired by fatigue. In narrative comments, students frequently described Step 1 to be a barrier to their development into effective clinicians, the traditional medical school curriculum to be a barrier to performance on Step 1, or both.

Conclusions

Medical students often prioritize Step 1 exam preparation over engaging with the standard preclinical curriculum, extracurricular opportunities, and activities to promote wellbeing. These findings have implications for the emphasis residency program directors place on single high-stakes standardized exams in the resident recruitment process.

## Introduction

The United States Medical Licensing Examination (USMLE) Step 1 score is one of the most commonly used data points to filter residency program applicants and plays a large role in determining medical students' competitiveness for medical specialties and specific residency programs [1,2]. Residency programs' focus on USMLE Step 1 scores has resulted in a standardized test "arms race" among medical students; this is reflected in a mean Step 1 score increase from 200 in 1993 to 230 in 2018 [3]. This was not the original intent of the USMLE Step 1. The National Board of Medical Examiners (NBME) originally conceived the USMLE Step exams as binary tests of a candidate's competence for medical licensure, stating as recently as 1990 that they reported numeric scores only out of "an obligation to provide examinees with knowledge of how their performances compare with passing scores" [4]. In spite of this, as mean Step 1 scores increase, applicants have their career opportunities defined by their performance on an increasingly small handful of questions. While several studies correlate USMLE Step 1 scores with performance on future standardized tests, data on whether Step 1 scores correlate with applicants’ subsequent clinical performance during residency are sparse, with the majority observing no association [5-11].

In a highly influential paper, Chen et al. argue that Step 1 preparation has become the primary goal of the first two years of medical school with detrimental implications for students' engagement with preclinical curricula with their focus being on content felt to be represented on the examination. Topics not prioritized by students include those related to newer competencies in health care such as medical ethics, professionalism, systems-based practice, bedside manners, and other topics perceived as "low-yield" for the exam [12]. They argue further that the intensely competitive Step 1 climate harms students' physical and mental wellbeing. However, there is little research validating these claims.

This study aimed to quantify the degree to which medical students choose not to engage with their regular preclinical curriculum (e.g. lectures and small group sessions) and extracurricular activities (e.g. research, community service, elective patient care, and leadership or healthcare advocacy) in order to focus on USMLE Step 1 preparation, as well learner-perceived effects of their preparation on their physical, social, and mental health.

## Materials and methods

An anonymous online survey was distributed to medical students at the University of Nebraska Medical Center (UNMC) who had taken the USMLE Step 1. UNMC is an accredited allopathic medical school with approximately 130 students per class; the mean and median Step 1 scores of the 2018-2019 class were 227 and 230.5; 64% of students matched to residencies in primary care, and 40% matched into in-state residency programs, the majority of whom matched to UNMC programs. The survey tool (Supplemental Appendix 1) included multiple choice and free response questions in the following domains: 1) demographic information, 2) time and financial resources devoted to USMLE Step 1 preparation, 3) degrees to which the respondent chose not to participate in UNMC’s preclinical curricula in order to prepare for USMLE Step 1, 4) degrees to which the respondent chose to participate in extracurricular activities in order to prepare for USMLE Step 1, 5) degrees to which USMLE Step 1 preparation adversely affected respondents’ physical, mental, and social/relational wellbeing, and 6) USMLE score and intended specialty. Numeric ranges for answer choices about medical school curriculum activities skipped were designed with input from medical students and medical education faculty. USMLE score was assessed as an ordinal variable in 10-point increments in lieu of asking for specific numeric scores, with the belief that this would increase the response rate.

Eligible medical students’ participation was solicited using the College of Medicine’s student e-mail listservs for the second, third, and fourth-year medical students. The initial survey request was distributed the last week of April 2020 (timed so that almost all second-year students would have taken USMLE Step 1 and received a score report, since the “dedicated study” timeframe concludes at the end of March in UNMC’s preclinical curriculum structure), and a single follow-up request was distributed one week later. To incentivize participation in the study, students were offered the opportunity to enroll in a lottery for one of twenty $20 gift cards for completing the survey.

Analysis was conducted in IBM’s Statistical Package for Social Sciences (SPSS) Statistics for Windows version 26 (IBM Corp., Armonk, NY, USA) using descriptive statistics and the chi-square, independent samples median test, one-way analysis of variance, and independent samples Mann-Whitney tests to assess associations between two or more categorical, ordinal, and continuous variables. We used p = 0.05 as the threshold for statistical significance and applied the Bonferonni correction for multiple comparisons as appropriate. Narrative feedback was categorized and reported using conventional content analysis.

This study was approved by the UNMC Institutional Review Board (IRB, #108-20-EX). To protect subject anonymity, all survey questions were optional, no potentially identifying demographic questions (i.e. demographics beyond gender) were included, and we committed in the IRB protocol to not report any subgroup analysis containing fewer than five subjects per category in any publication or presentation.

## Results

Description of the survey respondents

Of the 388 medical students invited to complete the survey, 211 (54%) submitted responses. Fifty percent of respondents were women. Ninety-one percent of respondents volunteered their most recent USMLE Step 1 score as a ranged ordinal variable, and 92% volunteered the primary residency specialty to which they had applied or planned to apply; these data, along with the median numbers of extracurricular experiences respondents completed in the first two years of medical school, are summarized in Table 1. Male respondents reported higher USMLE scores than their female peers (median response 231-240 vs 221-230 with mean ranks 107.8 vs 83.3; r=0.23 and p=0.002).

**Table 1:** Preclinical extracurricular experiences, USMLE Step 1 scores, and intended residency specialties ^a^Percentages given are of the total number of students who answered the question ^b^Internal medicine, family medicine, pediatrics, and obstetrics/gynecology ^c^Urology, orthopedic surgery, otolaryngology, oral and maxillofacial surgery, neurosurgery, and plastic & reconstructive surgery USMLE: United States Medical Licensing Examination, IQR: interquartile range

Experience type:	Median (IQR):
Research projects	1 (1 - 2)
Volunteering/community service	4 (2 - 6)
Elective patient care	1 (0 - 3)
Leadership & healthcare advocacy	1 (0 - 2)
USMLE Step 1 Score:	# of respondents (%)^a^
Greater than 250	29 (15)
241-250	36 (19)
231-240	42 (22)
221-230	34 (18)
211-220	24 (13)
210 or less	27 (14)
Intended specialty of residency	
Primary care ^b^	94 (49)
Surgical subspecialty ^c^	25 (13)
Other	72 (38)

Students invested significant time and money into Step 1 preparation, neither of which correlated with score outcome

Table 2 summarizes responses regarding time spent studying outside of class per week, the percentage of study time devoted specifically to USMLE Step 1 preparation, and the most common Step 1 study materials used for exam preparation. Students spent a mean $678 on Step 1 preparatory materials (SD $547, range $0-$3000); in total, the 388 students who completed the study reported spending $135,914 on USMLE Step 1 preparation materials. USMLE Step 1 scores did not correlate with average hours of study per week (p=0.10), percentage of study time devoted to USMLE Step 1 preparatory materials (p=0.13), total expenditure on Step 1 preparatory materials (p=0.40), or use of any particular study material (p>0.05 except for p=0.02 for more frequent use of USMLERx among students with higher scores, non-significant after Bonferroni correction for multiple comparisons).

**Table 2:** Preclinical study habits and choices of USMLE Step 1 study materials ^a^Percentages given are of the total number of students who answered the question USMLE: United States Medical Licensing Examination, NBME: National Board of Medical Examiners

Hours spent studying outside of class (weekly)	# of respondents (%)^a^
More than 80 hours	10 (5)
41-80 hours	59 (29)
21-40 hours	104 (51)
10-20 hours	21 (10)
Fewer than 10 hours	11 (5)
Percentage of study time specifically devoted to Step 1 preparation	
More than 80%	8 (4)
61-80%	10 (5)
41-60%	25 (12)
20-40%	61 (30)
Less than 20%	101 (49)
Choice of study materials	
UWorld USMLE Step 1 question bank	199 (94)
Pathoma	190 (90)
First Aid for the USMLE Step 1	183 (87)
Sketchy Micro	177 (84)
Sketchy Pharm	169 (80)
NBME Comprehensive Basic Science Assessments	135 (64)
Anki flashcards	118 (56)
Boards and Beyond	100 (47)
CramFighter	88 (42)
USMLERx	31 (15)
Osmosis	25 (12)
Firecracker	16 (8)
Kaplan USMLE Step 1 question bank	12 (6)
Doctors in Training	11 (5)

Students frequently skip curricular activities in order to study for USMLE Step 1

Table 3 describes the degree to which respondents chose not to participate in the standard UNMC preclinical curriculum in order to study for Step 1, including traditional preclinical lectures; small group sessions or other curricular activities devoted to medical ethics, healthcare policy, health systems science, or bias and disparities inequities in healthcare; sessions devoted to clinical skills training (e.g. history-taking, physical examination, clinical reasoning and decision-making, communicating effectively with patients, and ‘bedside manner’); and sessions involving real patients, their families, and/or standardized patients.

**Table 3:** Disengagement with the preclinical curriculum for the purpose of USMLE Step 1 exam preparation ^a^Percentages given are of the total number of students who answered the question USMLE: United States Medical Licensing Examination

Preclinical lectures skipped	# of respondents (%)^a^
More than 40	12 (6)
21 to 40	24 (12)
1 to 20	54 (27)
None	113 (56)
Sessions on medical ethics, healthcare policy, or bias and disparities in healthcare skipped	
More than 15	7 (4)
11 to 15	8 (4)
6 to 10	20 (10)
1 to 5	39 (19)
None	128 (63)
Sessions on basic clinical skills skipped	
More than 4	9 (5)
3 to 4	12 (6)
1 to 2	23 (11)
None	158 (78)
Sessions involving real patients, their families, and/or standardized patients skipped	
More than 4	5 (3)
3 to 4	5 (3)
1 to 2	5 (3)
None	187 (91)

Students’ USMLE scores were not significantly associated with the degree to which they skipped preclinical lectures or curricular activities devoted to medical ethics and health policy, basic clinical skills, or real or standardized patient encounters (p>0.05 for all). Similarly, these measures of curricular disengagement did not correlate with students’ plans to pursue careers in primary care, a surgical subspecialty, or other specialties (p>0.05 for all).

Students frequently forgo extracurricular opportunities in order to study for USMLE Step 1

During the first two years of medical school, students reported declining or choosing not to seek out a number of extracurricular activities in order to spend more time studying for Step 1. Students most often reported forgoing medical research projects (53%), followed by elective patient care activities such as participating in student-run free clinics (45%), community service activities such as volunteering at health screening fairs, health literacy events, shelters, and food banks (39%), and leadership or healthcare advocacy activities (38%).

Students who reported declining or choosing not pursue community service activities achieved higher USMLE Step 1 scores (median response 231-240 vs 221-230 with mean ranks 109.2 vs 88.4; r=0.19 and p=0.01); Step 1 scores were not associated with having foregone research, elective patient care, or leadership and advocacy activities. Planned entry into primary care, a surgical subspecialty, or another specialty was not associated with forgoing any type of extracurricular activity (p>0.05 for all).

Students identify USMLE Step 1 preparation as the cause of adverse effects on their behavior and wellbeing

Table 4 summarizes respondents’ reports of adverse effects they related to Step 1 exam preparation, broadly categorized into psychological, interpersonal, and behavioral consequences. Students’ responses to these questions did not vary based on their planned entry into a primary care, surgical subspecialty, or other residency program (p>0.05 for all). However, students had higher USMLE scores if they had not reported operating a motor vehicle while fatigued (median response 231-240 vs 221-230 with mean ranks 102.7 vs 78.9; r=0.19 and p=0.008), delaying or choosing to forgo seeking medical care (median response 231-240 vs 221-230 with mean ranks 105.9 vs 84.2; r=0.20 and p=0.007), delaying or forgoing major life events (median response 231-240 vs 221-230 with mean ranks 104 vs 79.7; r=0.2 and p=0.005), having relationship difficulties (median response 231-240 vs 221-230 with mean ranks 109.1 vs 76.4; r=0.29 and p<0.001), having feelings of anxiety or depression (median response 231-240 vs 221-230 with mean ranks 115.8 vs 83.6; r=0.29 and p<0.001), or having feelings of burnout (median response 241-250 vs 221-230 with mean ranks 125.1 vs 88.7; r=0.27 and p<0.001).

**Table 4:** Adverse effects of USMLE Step 1 preparation on dimensions of wellbeing ^a^Percentages given are of the total number of students who answered the question ^b^Defined in the question as “emotional exhaustion manifesting with detachment, cynicism, or blunted compassion, and accompanied by loss of satisfaction with or motivation in one’s role as a healthcare provider” USMLE: United States Medical Licensing Examination

	# of respondents (%)^a^
Psychological effects	
Experienced burnout ^b^	156 (79)
Experienced persistent or overwhelming feelings of anxiety or depression	120 (61)
Sought mental healthcare for anxiety, depression, or burnout	60 (31)
Interpersonal effects	
Missing family and significant other’s major life events (e.g. birth, marriage, hospitalization, or funeral)	58 (29)
Delaying or forgoing one’s own major family event (e.g. entering a long-term relationship, marrying, having a child, or taking parental leave to bond with a new child)	62 (32)
Having relationship difficulties or ending a relationship with a family member or significant other	77 (39)
Behavioral effects	
Delaying or forgoing one’s own medical care	87 (44)
Operating a motor vehicle while impaired by fatigue	53 (27)
Providing patient care compromised by fatigue	12 (6)
Using non-prescribed prescription stimulant medications to aid studying	8 (4)
Aware of a classmate using non-prescribed prescription stimulant medications to aid studying	59 (30)

Students recognize both the costs and value of Step 1 preparation, but the majority identify Step 1 preparation as having adverse influences on their education

A free response question solicited respondents’ thoughts about the costs and values of the Step 1 study process. Students submitted 53 responses, of which nine were wholly off-topic, principally opinions on the merits of various USMLE Step 1 preparation study strategies and grievances over the medical school’s perceived expectations-setting about the need to study for Step 1. We performed a conventional content analysis of the remaining 44 unique comments, identifying four main themes described in Table 5. While the single most common category of comment (n=13) was recognition of the exam preparation’s educational value, the majority of comments were critical: of these, students most frequently described negative impacts on their wellbeing (n=12) or identified the exam as a significant barrier to professional development, as a poor indicator of their clinical potential and/or performance, or both (n=12).

**Table 5:** Conventional content analysis of narrative comments on the costs and values of USMLE Step 1 exam preparation USMLE: United States Medical Licensing Examination

Theme	N	Representative Examples:
Step 1 studying was important preparation for the clinical years of medical school and residency	13	“While the problems with Step 1 are absolutely endless, they at least provided a basic framework to hang information off of- knowing what kind of basic science knowledge has relevance to the practice of medicine. This is contrasted to lectures from some basic scientists, who either do not know or do not have interest in making sure the information presented serves a role in informing the practice of medicine later in our careers” “I think that the score on Step 1 forced me to study far more in depth than I would have if it was pass/fail, and I processed/integrated information at a higher level- as a result I was FAR more prepared for clinical work” “While there are clearly mixed feelings about Step 1, I do believe that the time and focus spent studying for this exam forced me to learn, re-learn, and enhance my understanding of so many different topics. It was the time where I cemented a number of subjects/topics from the didactics that I simply did not have the ability to consolidate during the first 1.5 years”
Our preclinical curriculum interferes with Step 1 studying or posed a barrier to my career goals	12	“I wish we had an organized lecture/presentation outlining the long-term importance of Step 1 and what ramifications certain scores might have on our realistic opportunity to be accepted into certain specialties…. I wish someone had urged me to use Step-focused resources to study throughout my first 2 years, rather than using lectures which are often times NOT high yield” “Because [of] the impact Step 1 had on residency, I was only interested in learning that content during the first two years regardless of what was being taught in the lectures” “The course load was too much to study for Step 1 and we were told not to study for Step 1 during the first year of medical school because [the later preclinical curriculum] would be so great… had Step 1 been emphasized earlier on, and had we been encouraged to start studying from day 1, my score would probably not have been so terrible”
Step 1 studying adversely affected my mental or physical health or other aspects of my wellbeing	12	“I was honestly afraid I was not going to physically survive the first two years of medical school because I was so anxious about scoring well on Step 1…. The pressure to perform well on Step 1 exacerbated my depression. When I finally got my score and realized it wasn’t a good score, I didn’t even care because I was just shocked that I had survived” “I did not eat well and skipped several meals a week while preparing for USMLE Step 1. I did not exercise at all during dedicated preparation for USMLE Step 1. I did not see my significant other or family members for 3+ weeks at a time during the preclinical years, especially during dedicated preparation for USMLE Step 1” “The survey only asked about sacrifices made to volunteerism/community service, leadership/advocacy, direct patient care, and medical research activities… however, I also sacrificed wellness activities to study more for Step 1 (orchestra rehearsals, writing club sessions, tennis league) because I didn’t feel I could spare the time”
Step 1 studying is a barrier to learning how to be a good doctor or is not an adequate measure of students’ clinical potential	12	“I felt USMLE Step 1 was focused on a lot of things I never saw or used in actual clinical practice and I wasted a lot of time studying. It did help reinforce/consolidate some information, but I think that could’ve been accomplished in a different, less stressful manner.” “I spent too much time during my preclinical years memorizing intricate details of systems that don’t actually affect patient care. I frequently found that I had missed the “big picture” so often during my preclinical years that I felt ashamed of my medical knowledge during my clinical years. Even though I’ll graduate at the top of my class, I feel like I lack the meaningful medical background knowledge I need to care for patients that I should have solidly learned in my preclinical years (anatomy, relevant physiology). I regret that I spent so much time learning to do well on my exams instead of finding ways to make the clinically relevant information we learned ‘stick.’” “It was difficult to focus on the content of our lectures knowing that more weight was put on our Step 1 score when it comes to matching. However, I believe our clinical lectures would have helped us in preparing to become better and more well-rounded physicians than studying for Step 1 has.”

The survey concluded by asking students, “Setting aside the importance of USMLE Step 1 in the residency match process, had you focused less on Step 1 exam preparation during the first two years of medical school, how do you think that would have affected the quality of your medical training?” Students most frequently responded that they did not believe the quality of their training would have been much different (42%); of those who disagreed, most felt that their training would have probably (33%) or definitely (13%) been better, whereas 9% and 4% felt their training would probably or definitely been worse. Students with higher scores were more likely to feel that Step 1 exam preparation improved the quality of their medical training (median USMLE score range 221-230 for the responses ‘definitely would have been better’ and ‘probably would have been better’ versus 231-240 for the response ‘would not have been much different’, 241-250 for the response ‘probably would have been worse’ and >250 for ‘definitely would have been worse’; p<0.001).

## Discussion

This single-center survey of students who had completed their preclinical education and USMLE Step 1 board examination evaluated the educational, extracurricular, personal, and professional opportunity costs associated with Step 1 preparation. Medical students frequently choose not to participate in standard preclinical activities in order to prepare for Step 1, and this is particularly true for curricula focusing on medical ethics, healthcare policy, and bias/disparities in healthcare, topics that are not currently emphasized on Step 1. Many students forgo a range of extracurricular activities including research, community service, elective patient care experiences, and leadership/advocacy roles in order to prepare for Step 1. Students identified Step 1 preparation as contributing to high rates of anxiety, depression, and burnout, neglect of physical health and relationships, and engagement in unprofessional and potentially dangerous behaviors such as illicit prescription stimulant use and driving and providing patient care while impaired by fatigue. Finally, while some medical students perceive Step 1 preparation to have been an important part of their medical education, more perceived Step 1 to be a barrier to their development into effective clinicians, the traditional medical school curriculum to be a barrier to performance on Step 1, or both.

Students who scored higher perceived Step 1 as more beneficial to the quality of their training and reported less adversity during exam preparation. We offer two potentially concurrent interpretations of this finding: (1) recall bias, with students who performed better having a rosier recollection of their preparation, and/or (2) difference in adversity overcome, with students who were privileged with fewer external stressors or who had more time and resources to devote to exam preparation viewing their experience more favorably than students with greater outside life stressors or resource limitations. While the systemic environment is widely regarded as the primary contributor to medical student burnout, individual characteristics also influence how workload and level of stress are experienced. A recent review article identified differences in both demographics (e.g. higher prevalence among non-minority and female students) and life stressors (e.g. personal, relational and financial concerns) as potential individual-level correlates of burnout [13].

Our study has limitations. It was conducted at a single medical school with a median USMLE Step 1 score comparable to the national average, where historically nearly two-thirds of students match into primary care specialties, and may underestimate the prioritization of Step 1 and associated burdens shouldered by medical students at more competitive programs. Although we asked about students skipping these activities to quantify the impact, we believe this underrepresents the level of disengagement toward these topics as students may have chosen to be physically present at a lecture but review Step 1 practice questions rather than attend to the lecture’s content. Our findings are limited in that they do not clarify the exacerbated implications of USMLE exam preparation on international medical graduates, students from underrepresented in medicine (URiM) groups, and trainees from other marginalized backgrounds, which have been discussed in other recent works [14,15]. Finally, while we have described the extent to which students disengage from the UNMC curriculum in order to study for Step 1, our study was not designed to evaluate the adequacy of UNMC’s preclinical curriculum to train young physicians. One possible interpretation of this data is that UNMC students disengage from the standard curriculum not only because they recognize the importance of a competitive Step 1 score, but because they perceive UNMC’s preclinical curriculum to be critically deficient. However, UNMC is in good standing with the Liaison Committee on Medical Education (LCME), so we consider this less likely.

In this study, students with higher USMLE scores were more likely to be men, to have chosen not to participate in volunteerism/community service activities in order to prioritize exam preparation, and to view their Step 1 preparation experiences as more positive and more valuable. However, they did not report spending more time studying (either for Step 1 specifically or overall) during the first preclinical years, calling in to question whether a high Step 1 score is a useful surrogate for work ethic or “grit”. In other studies, higher USMLE Step 1 scores have correlated with male gender, URiM ethnicity, and “traditional” trainee age while failing to consistently predict evaluations of performance during internship [16-19]. While USMLE Step 1 scores do correlate with specialty board certification exam pass rates, trainees at substantial risk of failing these exams typically have Step 1 scores well below average [20,21].

In addition to whether higher step scores actually identify applicants with greater clinical potential, residency program directors (as the primary stakeholders driving the value of high USMLE Step scores) should consider the effect that the Step score arms races appear to have on student interest in participating in traditional medical school curricula and extracurricular opportunities, as well as the multitude of adverse effects on their future trainees’ wellbeing. Is the value added by a numeric Step 1 score worth recruiting interns who are dealing with high rates of anxiety, depression, and burnout, have normalized driving and practicing medicine while impaired, and who engage less with material on medical ethics, cultural competency, and health care disparities because those topics were not perceived to be “high-yield”? When residency directors bemoan that their house staff skip didactics to complete non-emergent patient care tasks, or spend more time in a patient’s electronic medical record than they do at the bedside, is it possible that valuing high performance on Step 1 might have selected for these bad habits? Over 80% of surveyed residency directors indicated that Pass/Fail reporting of Step 1 will result in the increased importance of Step 2 Clinical Knowledge (CK) scores, perhaps explaining why only 24.9% believed student wellbeing will improve [22]. The change to Pass/Fail reporting of Step 1 offers program directors an opportunity to meaningfully alleviate the burdens and maladaptive behaviors described here; however, this is unlikely if they merely choose to replace one high-stakes standardized exam with another.

## Conclusions

This study shows that medical students at a large Midwestern academic medical center frequently disengaged from planned preclinical curricula in order to prepare for Step 1, perceived the need for intense exam preparation had adverse effects on their wellness and behavior across multiple domains, and identified an overemphasis on Step 1 preparation as a barrier to being able to focus on becoming effective clinicians. How best to assess and select future physicians without emphasizing high Step scores is beyond the scope of this discussion. However, these results suggest that single “objective” metrics, including USMLE exams, ought to be de-emphasized in the future of residency selection. The planned transition to Pass/Fail reporting of USMLE Step 1 scores presents an opportunity for medical schools to re-center their preclinical curricula on mission-driven content, and for residencies to reboot their selection processes with new methods that are not only predictive of success during residency, but equitable and humane.

## Appendices

Survey Tool

PART 1: ASSESSMENT OF TIME AND RESOURCES SPENT STUDYING DURING THE PRECLINICAL YEARS

1. During the first 2 years of medical school, how many hours a week on average did you spend studying outside regularly scheduled classes?

A) Fewer than 10 hours

B) 10 to 20 hours

C) 21 to 40 hours

D) 41 to 80 hours

E) More than 80 hours

2. During the first 2 years of medical school, what proportion of your studying time did you spend studying outside resources explicitly intended for USMLE Step 1 preparation (e.g. Pathoma, Sketchy Micro or Pharma, First Aid, UWorld, Step 1 Anki decks, etc)?

A) Less than 20%

B) 20-40%

C) 41-60%

D) 61-80%

E) More than 80%

3. How much have you spent on USMLE Step 1 preparation materials (e.g. books, question banks, live or online courses, etc; NOT including registration fees)?  (Please enter a dollar value)

4. Which of the following resources did you use to study for the USMLE Step 1 (Select all that apply)?

UWorld question bank

Pathoma

Sketchy Micro

Sketchy Pharm

Sketchy Medical

First Aid for the USMLE Step 1

Boards and Beyond

NBME comprehensive basic science self-assessments

Cram fighter

USMLERx

Osmosis

Doctors In Training (DIT)

Kaplan

Firecracker

Anki

Other (please specify)

PART 2: ASSESSMENT OF CURRICULAR OPPORTUNITY COSTS

5. In total, how many preclinical lectures would you estimate you skipped to focus on USMLE Step 1 exam preparation?

A) None

B) 1 to 20

C) 21 to 40

D) 41 to 60

E) 61 to 80

F) 81 to 100

G) More than 100

6. In total, how many lectures, small group sessions, or other activities focused on training basic clinical skills (e.g. history-taking, physical examination, clinical reasoning and decision-making, communicating effectively with patients, or “bedside manner”) would you estimate you skipped to focus on USMLE Step 1 preparation?

A) None

B) 1 to 2

C) 3 to 4

D) 5 to 6

E) 7 to 8

F) 9 to 10

G) More than 10

7. In total, how many lectures, small group sessions, or other activities that involved real patients, their families, and/or standardized patients to focus on USMLE Step 1 preparation?

A) None

B) 1 to 2

C) 3 to 4

D) 5 to 6

E) 7 to 8

F) 9 to 10

G) More than 10

8. In total, how many lectures, small group sessions, or other activities concerning medical ethics, healthcare policy, health systems science, or bias/disparities in healthcare to focus on USMLE Step 1 preparation?

A) None

B) 1 to 5

C) 6 to 10

D) 11 to 15

E) 16 to 20

F) 21 to 25

G) More than 25

PART 3: ASSESSMENT OF EXTRACURRICULAR OPPORTUNITY COSTS

Note: for the questions below, define “project” or “activity” as a single effort rather than as individual sessions/instances (e.g. if you participated in a student-run health clinic and went to that clinic 8 times across during medical school, count that as one activity).

9. How many medical research projects (whether or not they yielded a poster, oral presentation, or publication) did you substantially contribute to during the first two years of medical school? (Enter a numeric value)

10. During the first two years of medical school, did you ever decline to participate in or consciously choose not to seek out a research project in order to focus on USMLE Step 1 preparation? (YES/NO)

11. How many volunteering/community service activities (e.g. providing free health screenings at community events, improving health literacy in schools or the community, volunteering at homeless shelters or food banks, etcetera) did you participate in during the first two years of medical school? (Enter a numeric value)

12. During the first two years of medical school, did you ever decline to participate in or consciously choose not to seek out a volunteering/community service activity in order to focus on USMLE Step 1 preparation? (YES/NO)

13. How many elective patient care activities (e.g. volunteering with nurses or phlebotomists in hospitals affiliated with the medical school, participating in a student-run free clinic) did you participate in during the first two years of medical school? (Enter a numeric value)

14. During the first two years of medical school, did you ever decline to participate in or consciously choose not to seek out an elective patient care activity in order to focus on USMLE Step 1 preparation? (YES/NO)

15. How many leadership or healthcare advocacy activities (e.g. organizing a student interest group, serving in a regional or national student organization, writing to or meeting with lawmakers or community members to advocate for effective healthcare policy) did you participate in during the first two years of medical school? (Enter a numeric value)

16. During the first two years of medical school, did you ever decline to participate in or consciously choose not to seek out a leadership or healthcare advocacy activity in order to focus on USMLE Step 1 preparation? (YES/NO)

Has your USMLE Step 1 preparation ever resulted in you...

17. Providing care for a patient you felt was compromised by your fatigue? (YES/NO)

18. Operating a motor vehicle while impaired by fatigue? (YES/NO)

19. Delaying or choosing to forgo seeking medical care? (YES/NO)

20. Using prescription stimulants (e.g. methyphenidate or dextroamphetamine) to enhance your studies (not including stimulants you are taking as prescribed to you to treat a medical condition) (YES/NO)?

21. Missing a family member or significant other’s important life event (e.g. birth, marriage, hospitalization, or funeral)? (YES/NO)

22. Having relationship difficulties or ending a relationship with a family member or significant other? (YES/NO)

23. Choosing to delay or forgo a major family event (e.g. getting into a long-term relationship, marriage, having a child, or taking parental leave to bond with a new child)? (YES/NO)

24. Having persistent or overwhelming feelings of anxiety or depression? (YES/NO)

25. Having symptoms of burnout (defined here as emotional exhaustion manifesting with detachment, cynicism, or blunted compassion, and accompanied by loss of satisfaction with or motivation in your role as health professional)? (YES/NO)

26. Seeking mental health care for feelings of anxiety, depression, or burnout? (YES/NO)

27. Are you aware of any of your classmates using prescription stimulants (except as prescribed to treat a medical condition) to enhance their studying for the USMLE Step 1?  (YES/NO)

28. Are you aware of any of your classmates expressing persistent or overwhelming feelings of anxiety or depression related to studying for the USMLE Step 1? (YES/NO)

29. Are you aware of any of your classmates expressing or demonstrating symptoms of burnout related to studying for the USMLE Step 1? (YES/NO)

30. Are you aware of any of your classmates seeking mental health care for feelings of anxiety, depression, or burnout related to studying for the USMLE Step 1? (YES/NO)

31. Setting aside the importance of USMLE Step 1 in the residency match process, had you focused less on Step 1 exam preparation during the first two years of medical school, how do you think that would have affected the quality of your medical training?

A) My training definitely would have been better overall

B) My training probably would have been better overall

C) The quality of my training would not have been much different

D) My training probably would have been worse overall

E) My training definitely would have been worse overall

Part 4: DEMOGRAPHIC DATA

A reminder: all survey responses are being collected anonymously and no potentially identifying responses will be reported in any publication or presentation.  Your responses and your participation in the study will be kept anonymous.

32. What is your gender?

A) Female

B) Male

C) Other OR prefer not to answer

33. What is the primary specialty you have applied to or are interested in applying to for residency? (Drop down list)

Anesthesiology

Dermatology

Emergency Medicine

Family Medicine

General Surgery

Internal Medicine

Neurology

Neurosurgery

Obstetrics/Gynecology

Ophthalmology

Orthopedic Surgery

Otolaryngology

Pathology

Pediatrics

Physical Medicine

Plastic Surgery

Psychiatry

Radiation Oncology

Radiology

Urology

Other (please specify)

34. What was your most recent USMLE Step 1 score?

A) Don’t know / my score has not yet been released

B) Less than 200

C) 200 to 210

D) 211 to 220

E) 221 to 230

F) 231 to 240

G) 241 to 250

H) More than 250

36.  Please feel free to share any comments you may have about this survey or how USMLE Step 1 preparation affected your preclinical training and/or wellbeing. (FREE RESPONSE)
